# Quinolizidine-Derived Lucanthone and Amitriptyline Analogues Endowed with Potent Antileishmanial Activity

**DOI:** 10.3390/ph13110339

**Published:** 2020-10-25

**Authors:** Michele Tonelli, Anna Sparatore, Nicoletta Basilico, Loredana Cavicchini, Silvia Parapini, Bruno Tasso, Erik Laurini, Sabrina Pricl, Vito Boido, Fabio Sparatore

**Affiliations:** 1 Dipartimento di Farmacia, Università degli Studi di Genova, Viale Benedetto XV, 3, 16132 Genova, Italy; tasso@difar.unige.it (B.T.); vito.boido@libero.it (V.B.); fabio.sparatore@alice.it (F.S.); 2 Dipartimento di Scienze Farmaceutiche, Università degli Studi di Milano, Via Mangiagalli 25, 20133 Milano, Italy; 3 Dipartimento di Scienze Biomediche Chirurgiche e Odontoiatriche, Università degli Studi di Milano, Via Pascal 36, 20133 Milano, Italy; loredana.cavicchini@unimi.it; 4 Dipartimento di Scienze Biomediche per la Salute, Università degli Studi di Milano, Via Mangiagalli 31, 20133 Milano, Italy; silvia.parapini@unimi.it; 5 Molecular Biology and Nanotechnology Laboratory (MolBNL@UniTS), DEA, Piazzale Europa 1, 34127 Trieste, Italy; erik.laurini@dia.units.it (E.L.); sabrina.pricl@dia.units.it (S.P.); 6 Department of General Biophysics, Faculty of Biology and Environmental Protection, University of Lodz, 90-236 Lodz, Poland

**Keywords:** *Leishmania tropica* and *infantum*, antileishmanial agents, lucanthone analogues, amitriptyline analogues, quinolizidine-derived compounds, molecular modelling studies

## Abstract

Leishmaniases are neglected diseases that are endemic in many tropical and sub-tropical Countries. Therapy is based on different classes of drugs which are burdened by severe side effects, occurrence of resistance and high costs, thereby creating the need for more efficacious, safer and inexpensive drugs. Herein, sixteen 9-thioxanthenone derivatives (lucanthone analogues) and four compounds embodying the diarylethene substructure of amitriptyline (amitriptyline analogues) were tested in vitro for activity against *Leishmania tropica* and *L. infantum* promastigotes. All compounds were characterized by the presence of a bulky quinolizidinylalkyl moiety. All compounds displayed activity against both species of *Leishmania* with IC_50_ values in the low micromolar range, resulting in several fold more potency than miltefosine, comparable to that of lucanthone, and endowed with substantially lower cytotoxicity to Vero-76 cells, for the best of them. Thus, 4-amino-1-(quinolizidinylethyl)aminothioxanthen-9-one (**14**) and 9-(quinolizidinylmethylidene)fluorene (**17**), with selectivity index (SI) in the range 16–24, represent promising leads for the development of improved antileishmanial agents. These two compounds also exhibited comparable activity against intramacrophagic amastigotes of *L. infantum*. Docking studies have suggested that the inhibition of trypanothione reductase (TryR) may be at the basis (eventually besides other mechanisms) of the observed antileishmanial activity. Therefore, these investigated derivatives may deserve further structural improvements and more in-depth biological studies of their mechanisms of action in order to develop more efficient antiparasitic agents.

## 1. Introduction

**Figure 1:**
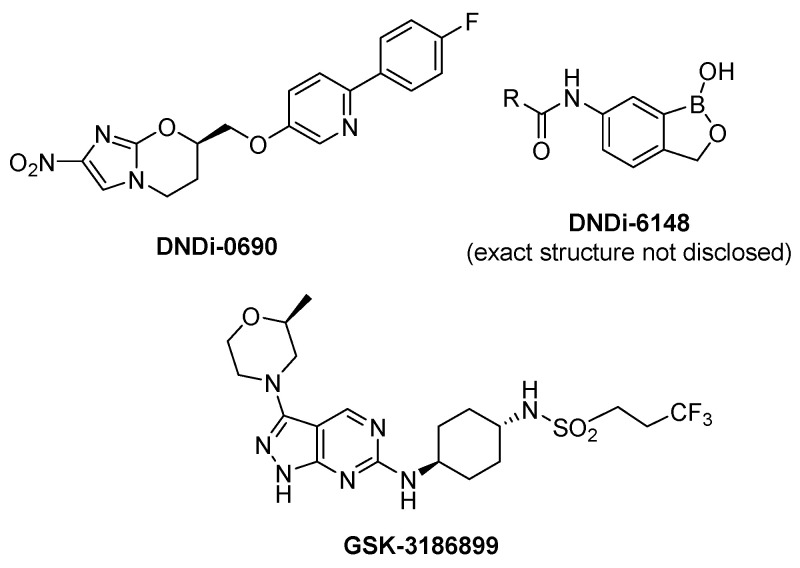
Antileishmanial drugs in clinical development.

Leishmaniases are neglected diseases that are endemic in many tropical and sub-tropical countries, leading annually to an estimated 700,000–1,000,000 new cases and 20,000–30,000 deaths [1]. Leishmaniases are caused by more than 20 species of protozoan parasites belonging to the genus *Leishmania*, which together with the genus *Trypanosoma,* belongs to the order *Trypanosomatidae*. Recent advances in the taxonomy, genetics, molecular and cellular biology and biochemistry of these organisms are well illustrated in two reviews [2,3]. The pathogen parasites (promastigotes) are transmitted to human and other mammalian hosts by the bites of infected female phlebotomine sandflies. In the mammalian host, the parasites differentiate into amastigote forms and affect skin, mucosa or internal tissues and organs to produce cutaneous (CL), muco-cutaneous (MC) and visceral (VL) leishmaniasis. The last form is fatal in absence of treatment. Current therapy is based on pentavalent antimonials; pentamidine; amphotericin B and its liposomal formulations used by parenteral route; and miltefosine, as the only oral agent. These drugs are burdened by heavy side effects, the occurrence of resistance and high costs, so that the need for novel, more efficacious, safe and inexpensive drugs is very stringent. Indeed, to meet this need, a number of studies are presently ongoing, exploring a very wide chemical space and also the possibility of the repositioning of known drugs. The structures of many interesting examples of investigational antileishmanial agents, able to hit different cellular targets, are illustrated in several reviews [4,5,6,7,8,9]. Interestingly, highly potent antileishmanial chemical classes, as the nitroimidazoles, the benzooxaboroles and the aminopyrazoles/pyrazolopyrimidines, have been identified, mainly within the Drugs for Neglected Diseases initiative (DNDi), Geneva, Switzerland. Orally active compounds from these series (such as DNDi-0690, DNDi-6148 and GSK-3186899) [10,11,12] are presently in clinical development (Figure 1).

**Figure 2:**
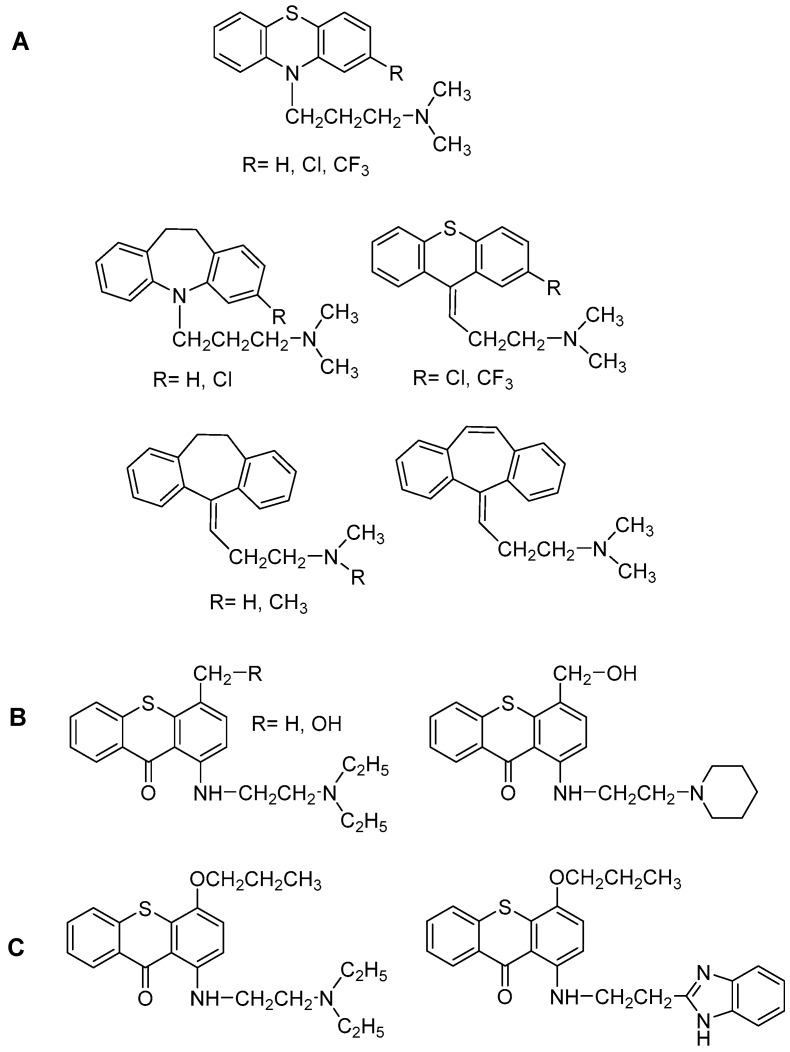
(**A**) Examples of basic derivatives of tricyclic systems displaying antileishmanial activity and inhibition of trypanothione reductase. (**B**) Basic derivatives of thioxanthene-9-one (lucanthone analogues) displaying antileishmanial activity. (**C**) Lucanthone analogues displaying dual inhibition of P-glycoprotein and cancer cell growth.

Additionally, many tricyclic antidepressant and antipsychotic drugs (and structurally related compounds) have been shown to display various degrees of activity against different species of *Leishmania*, and/or to inhibit enzymes (such as trypanothione reductase) playing essential roles in parasite development and virulence [13,14,15,16]. Among these, clomipramine and cyclobenzaprine have been recently repurposed for the treatment of visceral leishmaniasis [15,16]. All these compounds are characterized by different kinds of linear tricyclic systems, such as phenothiazine, iminodibenzyl, thioxanthene, dibenzocycloheptane and sulphur isosteric analogues, to which an aliphatic basic side chain is attached to the central ring (Figure 2A).

Thioxanthen-9-one is another tricyclic system whose derivatives exhibited antileishmanial activity, even featuring the basic side chain linked to a lateral ring (Figure 2B). Indeed, lucanthone, a drug largely used in the past for the treatment of schistosomiasis and presently (together with various analogues) under investigation as an antitumor agent, was shown to display activity against *L. major amazonensis* in tissue cultures a long time ago (1975) [17]. Although it was less powerful, this activity was also confirmed in vivo [18]. A few years later, this compound was also found to be active against intramacrophagic *L. tropica* amastigotes with IC_50_ = 0.93 µg/mL (2.47 µM) [19]. More recently (2011), through the screening of more than 4000 compounds, using a novel ex vivo splenic explant model system, an 1-(piperidinoethylamino)-analog of hycanthone (the active metabolite of lucanthone) was identified as a lead compound against *L. donovani*, with IC_50_ = 9.1 µM [20]. Hycanthone and its prodrug lucanthone are burdened with hepatic toxicity and mutagenicity [21,22,23], which were related to the presence or formation of a 4-hydroxymethyl group capable of alkylating the deoxyguanosine residue of DNA passing through the formation of a strongly electrophilic carbocation. Modification of the basic side chain and/or the introduction of substituents in position 6 of the thioxanthenone ring were shown to alter the mutagenicity, while retaining appreciable anti-schistosomal activity [24,25]. Even more, the replacement of the 4-methyl and 4-hydroxymethyl groups with other functional moieties limited the toxicological issues, as observed for the 4-propoxy analog of hycanthone (TXAI), investigated as an antitumor agent (Figure 2C) [26]. These data suggest that genetic and anti-parasitic activities of thioxanthenone derivatives may be dissociated from each other.

## 2. Results and Discussion

Thus, when pursuing our investigation on antiplasmodial [27,28,29] and antileishmanial [30,31] agents we deemed worthwhile the study of the antileishmanial activity of a set of lucanthone analogues with a modified substitution pattern, and of a few compounds embodying the diarylethene substructure of, e.g., chlorprothixene, amitriptyline and cyclobenzaprine. The studied compounds were characterized by the presence of the bulky quinolizidinylalkyl moieties, which were shown to improve the antiplasmodial and/or antileishmanial activity in the corresponding chloroquine and clofazimine analogs [30] and also in the set of 1-basic substituted 2-phenyl/benzyl benzimidazoles [31]. In position 4 of the thioxanthen-9-one nucleus, besides the methyl, a nitro or amino group was introduced, which were shown to improve the inhibitory activity in another field (against the lymphocytic leukemia P388 [32]). The considered compounds were obtained from our in-house library, having been synthesized and studied in the past as antimicrobial and anti-leukemia P388 agents [33,34]; as modulators of uptake and release of neurotransmitters [35,36,37]; and more recently, as dual inhibitors of cholinesterases and Aβ aggregation [38,39].

**Figure 3:**
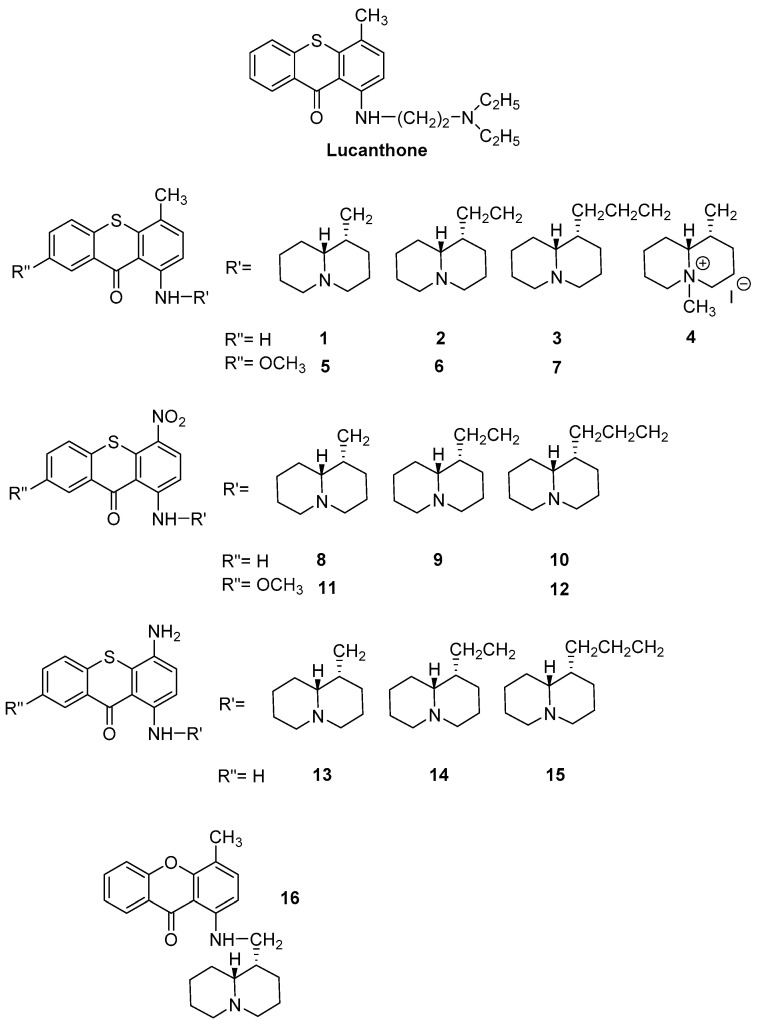
Investigated thioxanthen-9-one derivatives (lucanthone analogues) bearing quinolizidine-alkyl side chains.

**Figure 4:**
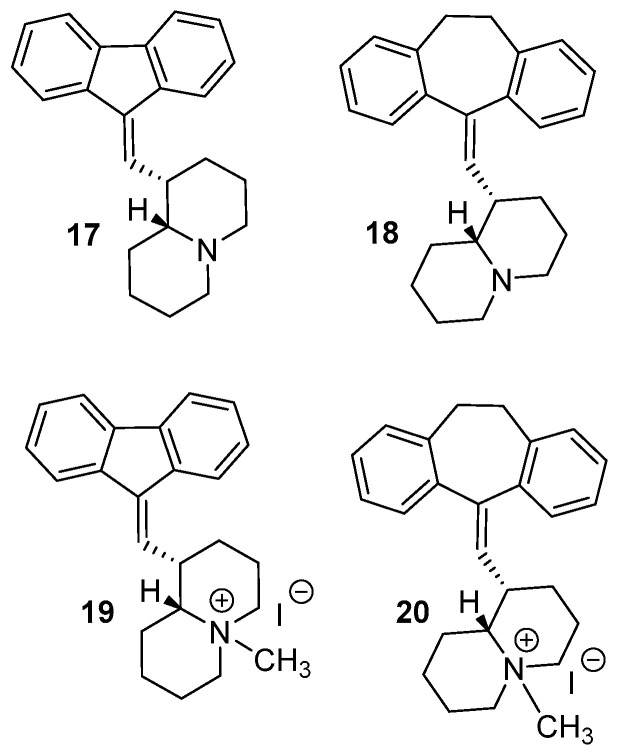
Investigated lupinylidene (quinolizidinyl-methylidene) derivatives of planar and corrugated tricyclic systems.

The structures of the presently investigated compounds are depicted in Figure 3 and Figure 4.

### 2.1. Chemistry

**Scheme 1:**
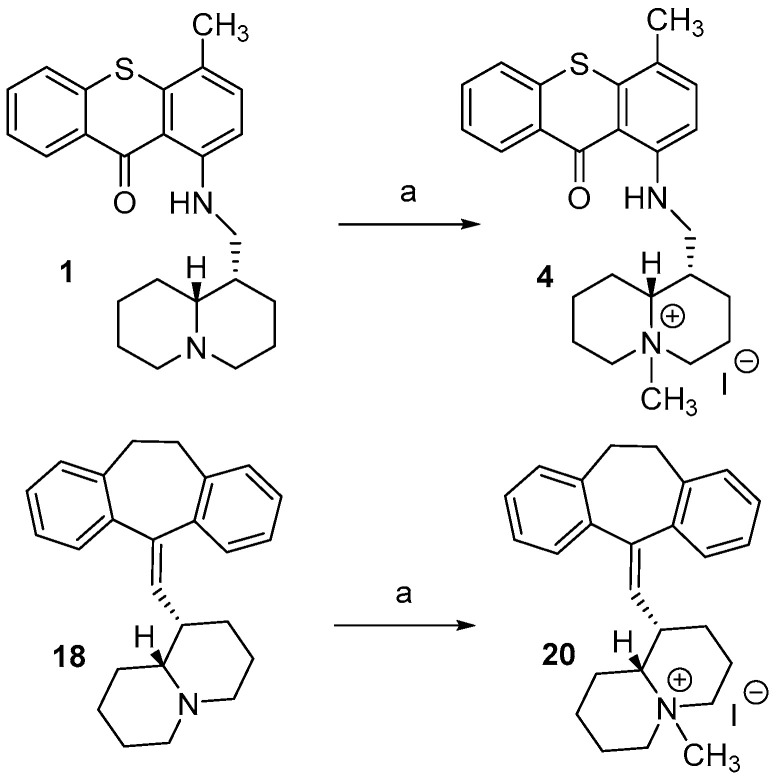
Reagents and conditions: (a) CH_3_I (excess), 24 h, r.t.

With the exception of **4** and **20**, all the compounds of Figure 3 and Figure 4 were previously described according to the following references: **1**–**3**, **5**–**10** and **13**–**15** [34]; **11**, **12** and **16** [38]; **17** and **18** [37]; **19** [33]. The novel compounds **4** and **20** were prepared by treating compounds **1** and **18** with methyl iodide (Scheme 1).

### 2.2. Biological Studies and SAR

#### 2.2.1. Antileishmanial Activity Against *L. tropica* and *L. infantum* Promastigotes

Compounds in Figure 3 and Figure 4 were tested in vitro against *Leishmania tropica* and *L. infantum* promastigotes using the MTT assay. Results are expressed as IC_50_ ± SD (µM) and reported in Table 1, together with the ratios between the IC_50_ of the reference drug (miltefosine) and that of each tested compound. All tested compounds displayed activity against both species of *Leishmania* and many of them exhibited IC_50_ less than 10 µM (65% and 50% versus *L. tropica* and *L. infantum*, respectively). Commonly, activity was higher for *L. tropica* than *L. infantum*. In comparison to miltefosine, all compounds were several–fold more potent, up to 17-fold against *L. tropica* and up to 9-fold against *L. infantum*. These results indicate that the introduction of a quinolizidinyl alkyl moiety on all the considered tricyclic systems is consistent with the expression of valuable antileishmanial activity, provided that, in the case of thioxanthenone, suitable substituents are present on position 4 and 7.

Among the thioxanthenone derivatives, compounds **9** and **13**–**15** exhibited potency (IC_50_ in the range 2.87–9.39 µM) comparable to that of lucanthone and 1-(piperidinoethyl)-4-hydroxymethylthioxanthen-9-one (the novel lead compound of Osorio et al. [20]), but differently from two of the others, they should not be associated with mutagenic activity because they lack groups potentially leading to alkylating species.

The elongation of the polymethylene linker exerted different effects, depending on the nature of the substituents, in position 4. Among the 4-methyl derivatives, the activity decreased with the increasing number of methylene groups in the side chain (IC_50_: from 3.36 to 8.89 µM and from 3.87 to 17.19 µM for the two *Leishmania* species), while among the 4-amino derivatives the activity remained practically unchanged (IC_50_ in the range 3–5 µM). In the set of 4-nitro derivatives, the activity varied for both species in an unaccountable way, with the worst IC_50_ values being for *n* = 1 (19.24 for *L. tropica* and 38.72 µM for *L. infantum*) and the best one being for *n* = 2 (IC_50_= 4.64 and 9.39 µM for *L. tropica* and *L. infantum*, respectively, Table 1). In other words, for a given number of CH_2_ in the side chain, the substitution of the 4-methyl with the 4-amino group, besides eliminating the risk of mutagenicity, always improved the activity of the relevant derivatives, while the presence of a 4-nitro moiety produced either a slight increase (*n* = 2) or a decrease (*n* = 1 and 3) of activity. In particular, the decrement of activity was very pronounced for *n* = 1, with IC_50_= 19.24 and 38.72 µM against *L. tropica* and *L. infantum*, respectively. Additionally, the introduction of a methoxy group in position 7 had variable effects on the activity, which was enhanced in the case of 4-nitro derivatives and decreased for the 4-methyl derivatives. The decreasing effects was striking when *n* = 2 (compound **6**), with IC_50_ = 28.35 µM and >46 µM for *L. tropica* and *L. infantum*, respectively. Thus compound **6** displayed the lowest activity among all tested compounds, although it was by far the least toxic (CC_50_ = 137 µM) on rat skeletal myoblast cells (L6), with a still valuable selectivity index (SI = 4.8 and ≤3) for the two *Leishmania* species (Reto Brun, personal communication to A.S.).

When comparing compounds **1** and **16**, it was observed that the antileishmanial activity was maintained with only modest loss of potency, indicating that even the exchange of the sulfur bridge with an oxygen atom did not modify significantly the thioxanthenone physico-chemical interactions with the *Leishmania* target(s), while it was able to abolish the antileukemic activity of lucanthone, as observed by Blanz and French [40]. On the contrary, the quaternarization of compounds **1** to give **4** produced a strong reduction of the antileishmanial activity, suggesting that the presence of a fixed charge, while hampering the usual target interactions, might shift the molecule towards a different cellular target. Supposing that the antileishmanial activity of the thioxanthenone derivatives could be related to one or some of the mechanisms previously described for the anti-schistosomal and anti-tumor activities of lucanthone (DNA intercalation, inhibition of nucleic acid biosynthesis, inhibition of topoisomerase II and apurinic endonuclease-1 [41], induction of autophagy and apoptosis [42], alteration of cholesterol biosynthesis and localization [43]), the quaternization of the quinolizidine nitrogen of **1** might produce the shift from some of these mechanisms to the inhibition of choline uptake, as it is known for common quaternary ammonium surfactants [44,45] and for the peculiar ammonium salts bearing bulky moieties on the charged head or on the lipophilic tail [46,47].

It is worth noting that compound **1** was shown in the past [33] to exhibit a large spectrum of antibacterial activity with MIC in the same micromolar range of IC_50_ against *Leishmania* species; f.i. MIC: 2.5 µg/mL = 6.63 µM against *Staphylococcus aureus* and *Bacillus subtilis*, and 1.25 µg/mL = 3.32 µM against *Mycobacterium tuberculosis*. Lucanthone also displayed antibacterial activity but with MIC 10-fold higher (30–60 µM) [48,49].

The four *lupinylidene derivatives* **17**–**20** embody the 1,1-diarylethene substructure that characterizes amitriptyline, cyclobenzaprime, chlorprothixene and other antidepressant and antipsychotic drugs. Like most of these drugs, compounds **17**–**20** exhibited antileishmanial activity in the low micromolar range; in particular, the 9-lupinylidene fluorene **17** (with IC_50_ = 3.5 and 4.35 µM against *L. tropica* and *L. infantum*, respectively) was 6/7-fold more potent than the lupinylidene dibenzocycloheptadiene **18** (IC_50_ = 24.25 and 25.21 µM), possibly for being endowed with a more appropriate lipophilicity. The quaternization of these compounds produced a levelling effect on activity, decreasing the potency of the former and increasing that of the latter, so that the corresponding methyl iodides **19** and **20** were almost equipotent.

The antileishmanial activity of the abovementioned drugs has been shown to be related to the inhibition of trypanothione reductase (TryR) in the parasite [50,51,52], but also to modulation of the immune response in the host. It is reasonable to suppose that also the tertiary compounds **17** and **18** act through inhibition of TryR; however, in the quaternized compounds **19** and **20**, other mechanisms might modulate or replace the former, as discussed above for quaternized thioxanthenone **4**. Interestingly, the tertiary amitriptyline analog **18** (compound **17** was not tested) was shown in the past [35] to inhibit the choline uptake into rat brain synaptosomes at 1 µM concentration, at which amitriptyline was still completely ineffective. The inhibition of choline transport across the synaptosome membrane might be expected in some measure also at the level of the parasite cell membrane in an interplay with the intracellular TryR inhibition. With the quaternization of **18** to **20**, cell penetration and consequent TryR inhibition could be strongly reduced, but the basal activity on choline transport could be improved.

Lupinylidene fluorene **17** was not investigated as an inhibitor of choline uptake, but it was shown to display a large spectrum of anti-microbial activity with outstanding potency against *Mycobacterium tuberculosis H37Ra* (MIC = 0.49 µM) very close to that of isoniazide (MIC: 0.14–0.28 µM) [33]. The quaternization of **17** to **19** strongly affected the antimycobacterial activity (MIC = 83.9 µM) quite probably by reducing the cell wall crossing capability.

Even if the definition of the mechanism of the antileishmanial activity of the tested compounds is beyond the scope of this exploratory work, one cannot overlook the previous observation that lucanthone [53], the quinolizidinylalkylamino thioxanthenones [38] and the lupinylidene dibenzocycloheptadiene [39] inhibit AChE, and particularly BChE, with IC_50_ in the low micromolar and sub-micromolar range. The availability of choline for building up the phosphatidylcholine, the main component of *Leishmania* promastigote membranes [54,55], might be compromised by the inhibition of cholinesterases. Cholinesterases are known to be present even in non-motile unicellular organisms, where besides or instead of the hydrolytic function, they may play non-classical roles that are fundamental for cell survival [56,57,58]. The inhibition of cholinesterases has been recently claimed as another mechanism of action for some antileishmanial agents extracted from several plants [59,60,61], and the present results add further support to this hypothesis.

#### 2.2.2. Cytotoxicity 

To identify new potential candidates for the development of safe and effective antileishmanial drugs, the cytotoxicities of representative (most effective or structurally peculiar) compounds (**1**, **2**, **14**, **17**, **19** and **20**) were evaluated in a Vero-76 cell line. The cytotoxicity of lucanthone was also tested for comparison. The results in Table 2 show that all compounds exhibited CC_50_ higher than their corresponding IC_50_ values. The relevant selectivity index (SI)—a parameter that quantifies the preferential antileishmanial activity of a compound in relation to mammalian cell toxicity (CC_50_/IC_50_)—is tabulated in Table 2. As can be seen from this Table, the 4-methyl-thioxanthen-9-ones (**1**, **2**, and lucanthone) exhibited, as expected, the highest cytotoxicity among the tested compounds. It is worth noting that compounds **1** and **2** were somewhat less toxic than lucanthone, suggesting a possible toxicity-lowering effect of the cumbersome quinolizidinylalkyl side chain in comparison to an open chain substituent. The replacement of the 4-methyl substituent with an amino group in compound **14,** while preserving good antileishmanial activity, led to a further substantial decrease of cytotoxicity, with a resultantly safer profile (SI = 16.2 and 16.9 for the two *Leishmania* species). 

On the other hand, the lupinylidene tricyclic derivatives **17**, **19** and **20** (the amitriptyline analogues, broadly speaking) displayed the lowest cytotoxic effects, with CC_50_ values in the range 80–90 µM, in the presence of either tertiary or quaternized nitrogen. However, taking into account the effects of quaternization on the activity, only the tertiary 9-lupinylidene fluorene **17** displayed a quite valuable SI value for both *Leishmania* species (23.9 and 16.2, respectively), resulting the most promising antileishmanial candidate among the whole set of studied compounds.

The cytotoxic concentration (CC_50_) against THP-1 differentiated into macrophages (Table 3), used for testing the activity against the amastigote stage, is also reported. Interestingly, compounds **1**, **14**, **17** and lucanthone showed a SI trend against THP-1 cells (Table 3) comparable to that against Vero-76 cells.

It is additionally observed that, based on the results of their previous testing for antileukemic activity [34], only a low to moderate level of in vivo toxicity should be expected. Indeed, no mortality was observed even when compounds **1**, **5**, **6**, **9** and particularly the amino derivatives **13**–**15** were injected i.p. at doses up to 200–350 mg/kg, once a day for five consecutive days, in a group of six mice previously inoculated with leukemia P388 cells. Particularly, for compound **14** no mortality was observed at a dose of 260 mg/kg (638 µmol/kg), and therefore this compound represents an interesting lead for improved lucanthone analogues.

#### 2.2.3. Antileishmanial Activity against L. Infantum Amastigotes

Finally, for a better idea of their real value as antileishmanial agents, compounds **14** and **17**, displaying the most promising activity against promastigote stage and the highest SI values, together with lucanthone and the corresponding quinolizidine analog **1**, were tested against the intramacrophagic amastigote stage of *L. infantum*. The results indicate that the activity observed against *L. infantum* promastigotes is conserved (lucanthone and compound **1**) or even improved (**14** and **17**) against the corresponding amastigote stage (Table 3). 

### 2.3. Molecular Modelling Studies 

**Figure 5:**
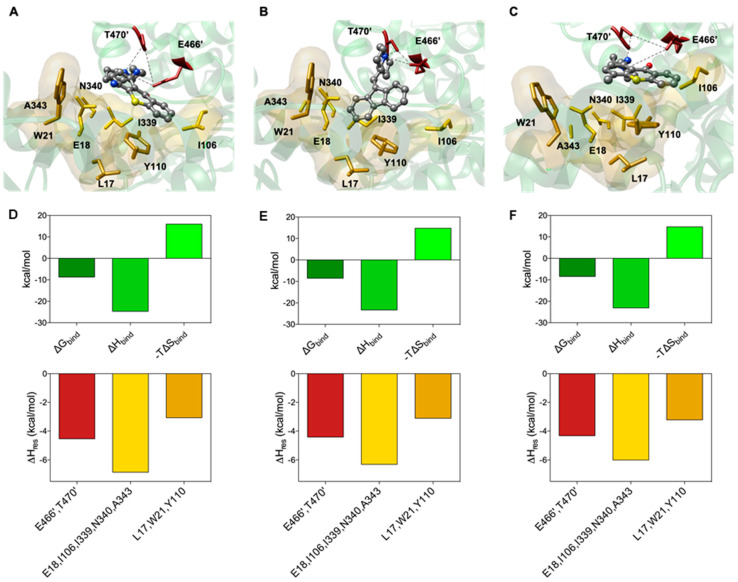
(top panel) Details of compounds **14** (**A**), **17** (**B**) and lucanthone (**C**) in the binding pocket of TryR. Compounds are shown as atom-colored sticks-and-balls (C, grey, N, blue, O, red). The side chains of the mainly interacting TryR residues are depicted as colored sticks and labeled as following: E466′ and T470′, firebrick; E18, W21, I339, N340 and A343, gold; L17, I106 and Y110, goldenrod. The hydrophobic pockets are also highlighted by their transparent van der Waals surface. Hydrogen atoms, water molecules, ions and counterions are omitted for clarity. (bottom panel) Calculated free energy of binding (ΔG_bind_, forest green), and enthalpic (ΔH_bind_, lime green) and entropic (−TΔS_bind_, chartreuse) components (upper row) and PRBFED of the main involved amino acids (bottom row) of TryR in complex with **14** (**D**), **17** (**E**) and lucanthone (**F**).

Amitriptyline and other related compounds have been identified as plausible inhibitors of the trypanothione reductase (TryR), an essential enzyme belonging to the antioxidant machinery of parasitic *Leishmania* [62,63]. TryR is a homodimer and it is active only in this aggregated form. Given some structural properties in common, we reasoned that the most potent compounds of this series, **14** and **17**, can exert their antileishmanial properties (mainly or besides other mechanisms) by efficiently binding TryR and consequently blocking its activity. In order to test our hypothesis, molecular dynamics (MD) simulations were performed on the corresponding complexes with TryR to shed light on the binding mechanisms of these compounds against their putative parasitic target (Figure 5). To validate our procedure and for comparison purposes, we applied the same computational procedure also to TryR in complex with lucanthone (Figure 5) and amitriptyline (Figure S1).

A putative binding site for these compounds was initially recognized on TryR following a consolidated protocol [64,65,66]. Through the MM/PBSA (molecular mechanics/Poisson–Boltzmann surface area) approach [67], we calculated each inhibitor/enzyme free energy of binding (ΔG_bind_) and its enthalpic and entropic components (ΔH_bind_ and -TΔS_bind_, respectively). The obtained values are in good agreement with their antileishmanial activity (Figure 5D–F and Table S1) yielding the following TryR affinity ranking: **14** (ΔG_bind_ = −8.73 kcal/mol) < **17** (ΔG_bind_ = −8.54 kcal/mol) <= lucanthone (ΔG_bind_ = −8.45 kcal/mol) << amitriptyline (ΔG_bind_ = −7.46 kcal/mol). Interestingly, all compounds share a common thermodynamics pattern; actually, their binding is robustly enthalpy driven characterized by favorable electrostatic and van der Waals interactions. On the other hand, the entropic components penalize the binding, as often detected in cases of small molecule/protein complexes. The precise binding mechanism and the specific ligand/protein interactions were elucidated through the per-residue binding free energy deconvolution (PRBFED) of the enthalpic terms (ΔH_res_). The PRBFED analysis allowed us to identify the main aminoacid residues of TryR involved in the putative binding pocket (Figure 5D–F, Figure S1 and Table S2).

The most peculiar interaction is definitively performed by the charged nitrogen atom present in all four of the compounds. Indeed, this protonated tertiary amine group is involved in a virtuous, interactive triangle with the side chain of the second monomer of TryR residues E466′ and T470′ through stable hydrogen bonds and a salt bridge. The length and the rigidity of the spacer between the nitrogen atom and the tricyclic moiety of the inhibitor can affect the efficiency of these interactions. In effect, the ylidenepropyl-amino spacer of amitriptyline is not able to provide the optimal addressing of the charged group towards the side chain of E466′ and T470′ with respect to the bulkiest quinolizidine moiety of **14** and **17** or the most flexible amino-ethyl spacer of lucanthone; accordingly, its ΔH_res_ values resulted the less favorable of the series (Figure 5D–F, Figure S1, and Table S2). On the other hand, it is already established that the dibenzocycloheptene ring of the amitriptyline can be aptly accommodated in the so-called TryR hydrophobic wall formed by residues L17, W21 and Y110 [62,63]. Our MD simulation confirmed this data (Figure S1) and our computational analysis allowed us to find other important TryR residues to improve the van der Waals interactions in this specific hydrophobic region (Figure S1). As listed in Table S2, the side chains of residues E18, I106, I339, N343 and A343 also had contributions to stabilizing the binding with the enzyme. It is worth to mention here that also the tricyclic scaffold of the other compounds can be encased in the same protein region with even better performance (Figure 5D–F, Table S2). In the case of compound **17**, this could be expected since the fluorene ring is very similar to the dibenzocycloheptene moiety, but for the thioxanthenone derivatives **14** and lucanthone this might not have been so obvious. Instead, our MD approach showed that both compounds can share a very similar TryR binding mode with the lupinylidene and amitriptyline derivatives. Finally, compound **14** was the best TryR binder and the amino substitution (-NH_2_) in position 4 of the thioxanthenone ring of 14 played an important role. Actually, this amino group can establish a further polar interaction with the side chain of E18 leading to a slight yet significative improvement of its binding capability, as shown in Figure 5D and Table S2.

## 3. Materials and Methods

### 3.1. Chemistry

#### 3.1.1. General Information

Chemicals, solvents and reagents used for the syntheses were purchased from Sigma-Aldrich or Alfa Aesar (Milan, Italy), and were used without any further purification. Melting points (uncorrected) were determined with a Büchi apparatus (Milan, Italy). ^1^H NMR and ^13^C NMR spectra were recorded with a Varian Gemini-200 spectrometer in CDCl_3_; the chemical shifts were expressed in ppm (δ), coupling constants (J) in Hertz (Hz). Elemental analyses were performed on a Flash 2000 CHNS (Thermo Scientific, Milan Italy) instrument in the Microanalysis Laboratory of the Department of Pharmacy, University of Genova. Q = quinolizidine ring; Ar= aromatic.

#### 3.1.2. General Procedure for the Synthesis of Quaternary Ammonium Iodides (**4** and **20**)

The compounds **1** [34] and **18** [37] (0.146 mmol) were reacted with iodomethane (0.5 mL, 8 mmol) at r.t. for 24 h with stirring. The reaction mixture was added with dry Et_2_O, and the collected compound was washed with dry Et_2_O affording the title quaternary ammonium salt.

(1*S*,9a*R*)-5-methyl-1-{[(4-methyl-9-oxo-9*H*-thioxanthen-1-yl)amino]methyl}-decahydroquinolizin-5-ium iodide (**4**): orange crystals; yield: 92%. m.p. 147–150 °C (Et_2_O an.); ^1^H NMR (200MHz, CDCl_3_): δ = 9.36 (s, NH–Ar, collapses with D_2_O); 8.40 (d, *J* = 9.2, 1 ArH), 7.43–7.20 (m, 4 ArH), 6.72 (d, *J* = 9.2, 1 ArH), 4.21–3.00 (m, 8H of Q and 3.51, s, CH_3_-N of Q), 2.95–2.83 (m, 1H, of Q), 2.40–1.40 (m, 9H of Q and 2.25, s, CH_3_-Ar superimposed); ^13^C NMR (50 MHz, CDCl_3_): 182.4, 149.4, 137.1, 135.1, 134.7, 130.9, 128.6, 128.0, 125.1, 124.4, 119.7, 112.3, 107.3, 67.9, 65.0, 51.5, 49.8, 44.6, 31.7, 20.4, 19.7, 19.0, 18.7, 18.0. Anal. calcd for C_25_H_31_IN_2_OS: C 56.18, H 5.85, N 5.24, S 6.00, found: C 56.09, H 6.15, N 5.15, S 6.39.

(1*S*,9a*R*)-1-[(10,11-dihydro-5*H*-dibenzo[a,d][7]annulen-5-ylidene)methyl]-5-methyl-decahydroquinolizin-5-ium iodide (**20**): pale yellow crystals; yield: 98%. m.p. 283–286 °C (Et_2_O an.); ^1^H NMR (200MHz, CDCl_3_): δ = 7.50–6.97 (m, 8 ArH), 6.58–6.40 (m, 1H, HC=), 3.58–3.20 (m, 2Hα near N of Q and 3.36, s, CH_3_-N of Q), 3.17–2.63 (m, 4H, 2CH_2_-Ar), 2.36–1.00 (m, 14H of Q); ^13^C NMR (50 MHz, CDCl_3_): 139.8, 138.7, 138.6, 135.6, 135.4, 129.1, 128.9, 128.5, 128.1, 126.7, 126.6, 124.8, 124.6, 64.7, 64.4, 56.1, 38.0, 37.0, 32.9, 30.8, 29.7, 24.4, 23.7, 23.5, 20.5, 20.1. Anal. calcd for C_26_H_32_IN: C, 64.34; H, 6.64; N 2.89. Found: C, 64.27; H, 7.00; N 2.70.

### 3.2. Biological Tests

#### 3.2.1. Antileishmanial Activity

Promastigote stage of *L. infantum* strain MHOM/TN/80/IPT1 (kindly provided by Dr. M. Gramiccia, ISS, Roma) and *L. tropica* (MHOM/SY/2012/ISS3130) were cultured in RPMI 1640 medium (EuroClone) supplemented with 10% heat-inactivated fetal calf serum (EuroClone, Milan Italy), 20 mM Hepes and 2 mM *L*-glutamine at 24 °C. To estimate the 50% inhibitory concentration (IC_50_), the MTT (3-[4.5-dimethylthiazol-2-yl]-2.5-diphenyltetrazolium bromide) method was used [68,69]. Compounds were dissolved in DMSO and then diluted with medium to achieve the required concentrations. Drugs were placed in 96 wells round-bottom microplates and seven serial dilutions made. Miltefosine was used as reference anti-*Leishmania* drug. Parasites were diluted in complete medium to 5 × 10^6^ parasites/mL and 100 μL of the suspension was seeded into the plates, incubated at 24 °C for 72 h and then 20 µL of MTT solution (5 mg/mL) was added into each well for 3 h. The plates were then centrifuged, the supernatants were discarded and the resulting pellets were dissolved in 100 µL of lysing buffer consisting of 20% (*w/v*) of a solution of SDS (Sigma), 40% DMF (Merck, Milan Italy) in H_2_O. The absorbance was measured spectrophotometrically at a test wavelength of 550 nm and a reference wavelength of 650 nm. The results are all expressed as IC_50_, which is the dose of compound necessary to inhibit parasite growth by 50%; each IC_50_ value is the mean of separate experiments performed in duplicate.

#### 3.2.2. In Vitro Intracellular Amastigote Susceptibility Assays

THP-1 cells (human acute monocytic leukemia) were maintained in RPMI supplemented with 10% FBS (EuroClone), 50 µM 2-mercaptoethanol, 20 mM Hepes and 2 mM glutamine, at 37 °C in 5% CO_2_. For *Leishmania* infections, THP-1 cells were plated at 5 × 10^5^ cells/mL in 16-chamber Lab-Tek culture slides (Nunc, Milan, Italy) and treated with 0.1 µM phorbol myristate acetate (PMA, Sigma) for 48 h to achieve differentiation into macrophages. Cells were washed and infected with metacyclic *L. infantum* promastigotes at a macrophage/promastigote ratio of 1/10 for 24 h. Cell monolayers were then washed and incubated in the presence of test compounds for 72 h. Slides were fixed with methanol and stained with Giemsa. The percentages of infected macrophages among treated and non-treated cells were determined by light microscopy [70].

#### 3.2.3. Cell Cytotoxicity Assays

THP-1 cells were plated at 5 × 10^5^ cells/mL in 96 wells flat bottom microplates and treated with 0.1 µM PMA for 48 h to achieve differentiation into macrophages. Cells were then treated with serial dilutions of test compounds and cell proliferation evaluated using the MTT assay described for promastigotes. The results are expressed as CC_50_, which is the dose of compound necessary to inhibit cell growth by 50%.

Vero-76 cells (ATCC CRL 1587 *Cercopithecus Aethiops*) were seeded at an initial density of 4 × 10^5^ cells/mL in 24-well plates, in culture medium (Dulbecco’s modified eagle’s medium (D-MEM) with L-glutamine, supplemented with fetal bovine serum (FBS), 0.025 g/L kanamycin). Cell cultures were then incubated at 37 °C in a humidified, 5% CO_2_ atmosphere in the absence or presence of serial dilutions of test compounds. Cell viability was determined after 48–96 h at 37 °C by the Crystal violet staining method. The results are expressed as CC_50_, which is the concentration of compound necessary to inhibit cell growth by 50%. Each CC_50_ value is the mean and standard deviation of at least three separate experiments performed in duplicate.

### 3.3. Computational Methods

The 3D structure of the TryR from *Leishmania infantum* was obtained starting from the available Protein Data Bank file (pdb code: 2JK6 [71]) and optimized following a procedure previously described [64,65,66]. The optimized structures of the new tested compounds were docked into the putative binding pocket using Autodock 4.2.6/Autodock Tools1.4.61 [72]. The resulting complex was further energy minimized to convergence. The intermolecular complex was then solvated by a cubic box of TIP3P water molecules [73] and energy was minimized using a combination of molecular dynamics (MD) techniques [64,65,66]. Ten nanosecond molecular dynamics (MD) simulations at 298 K were then employed for system equilibration, and further, 50 ns MD simulations were run for data production. Following the MM/PBSA approach [67] each binding free energy value (ΔG_bind_) was calculated as the sum of the electrostatic, van der Waals, polar solvation, nonpolar solvation, (ΔH_bind_) and entropic contributions (TΔS_bind_). The PRBFED analysis was carried out using the molecular mechanics/generalized Boltzmann surface area (MM/GBSA) approach [74] and was based on the same snapshots used in the binding free energy calculation. All simulations were carried out using the pmemd and pmemd.CUDA modules of Amber 18 [75], running on our own CPU/GPU calculation cluster. Molecular graphics images were produced using the UCSF Chimera package (v.1.14) [76]. All other graphs were obtained using GraphPad Prism (v. 6.0, GraphPad, La Jolla, CA, USA).

## 4. Conclusions

Sixteen 9-thioxanthenone derivatives (lucanthone analogues) and four compounds embodying the diarylethene substructure of amitriptyline (amitriptyline analogues) were tested in vitro for activity against *Leishmania tropica* and *L. infantum* promastigotes, and in a few cases also against intramacrophagic amastigotes of *L. infantum*. All compounds were characterized by the presence of a bulky quinolizidinylalkyl moiety, while differing for the tricyclic system to which the basic chain was connected. All compounds displayed activity against both species of *Leishmania* and most of them exhibited IC_50_ values lower than 10 µM, and were many-fold more potent than miltefosine. The six best compounds (**1**, **9**, **13**–**15** and **17**) displayed potency comparable to that of lucanthone (IC_50_ = 2.5 and 3.5 µM for the two *Leishmania* species), but their cytotoxicity versus the Vero 76 cells was always lower, with significant improvement of SI from 4.8–3.5 (lucanthone) to 16.2–16.9 and 23.6–19.2 for compounds **14** and **17**, respectively. These compounds exhibited comparable activity (and selectivity against THP-1 cells) against intramacrophagic amastigotes of *L. infantum*, and thus represent promising, structurally distinct leads for the development of improved antileishmanial agents. Docking studies suggest that the antileishmanial activity of compounds **14** and **17** may be related to the inhibition of trypanothione reductase, as is the case for other tricyclic compounds. However, lucanthone and the tested compounds were previously shown to potently inhibit the AChE, and particularly the BChE; thus, it is now put forward that this inhibitory property may have a notable additional role in the antiparasitic mechanism, either by reducing the availability of choline to build up the main component of promastigote membrane, or by inhibiting other non-classical functions of cholinesterases in the unicellular organisms.

## Figures and Tables

**Table 1:** In vitro data on antileishmanial activity against *Leishmania tropica* and *L. infantum* promastigotes of lucanthone and compounds **1**–**20**.

Compd.	IC_50_ (µM) ^a^ *L. tropica*	Ratio ^b^ IC_50_ Miltef./IC_50_ Compd.	IC_50_ (µM) ^a^ *L. infantum*	Ratio ^b^ IC_50_ Miltef./IC_50_ Compd.
**Lucanthone**	2.57 ± 1.03	16.8	3.50 ± 1.17	8.9
**1**	3.3 6 ± 1.53	12.9	3.87 ± 1.53	8.1
**2**	6.27 ± 2.75	6.9	10.60 ± 3.78	2.95
**3**	8.89 ± 3.02	4.87	17.19 ± 1.31	1.8
**4**	17.83 ± 5.61	2.4	13.00 ± 2.80	2.4
**5**	8.16 ± 5.23	5.3	8.99 ± 3.86	3.5
**6**	28.35 ± 14.34	1.5	>46	<0.7
**7**	12.00 ± 5.24	3.6	19.66 ± 5.79	1.6
**8**	19.24 ± 9.33	2.25	38.72 ± 7.67	0.8
**9**	4.64 ± 1.12	9.3	9.39 ± 4.34	3.3
**10**	11.05 ± 3.30	3.9	22.27 ± 3.30	1.4
**11**	6.35 ± 1.46	6.8	13.01 ± 0.97	2.4
**12**	7.39 ± 2.31	5.85	7.89 ± 2.41	3.95
**13**	2.87 ± 0.43	15.1	5.23 ± 1.83	6.0
**14**	3.80 ± 1.82	11.4	3.63 ± 0.78	8.6
**15**	3.72 ± 1.66	11.6	4.22 ± 1.95	7.4
**16**	6.56 ± 3.08	6.6	8.23 ± 1.51	3.8
**17**	3.49 ± 1.02	12.4	4.35 ± 0.37	7.2
**18**	24.25 ± 3.58	1.8	25.21 ± 1.92	1.24
**19**	10.52 ± 2.69	4.1	16.42 ± 0.59	1.90
**20**	7.21 ± 3.52	6.0	8.71 ± 0.68	3.58
**Miltefosine**	43.26 ± 11.36	1.0	31.26 ± 10.45	1.0

^a^ The results are expressed as IC_50_ ± SD of at least three different experiments performed in duplicate. ^b^ Ratios between the IC_50_ of miltefosine and that of each compound against *L. tropica* or *L. infantum*.

**Table 2:** In vitro cytotoxicity data against Vero-76 cells and selectivity index (SI) values for selected antileishmanial compounds of Table 1.

Compd.	CC_50_^a^ (µM) Vero-76	IC_50_^b^ (µM)		S.I. ^c^	
		*L. tropica*	*L. infantum*	*L. tropica*	*L. infantum*
**Lucanthone**	12.4 ± 2.1	2.57 ± 1.03	3.50 ± 1.17	4.8	3.5
**1**	23.5 ±1.8	3.36 ± 1.53	3.87 ± 1.53	7.0	6.6
**2**	22.9 ± 2.3	6.27 ± 2.75	10.60 ± 3.78	3.7	2.2
**14**	61.4 ± 2.6	3.80 ± 1.82	3.63 ± 0.78	16.2	16.9
**17**	83.4 ± 3.0	3.48 ± 1.02	4.35 ± 0.37	23.9	19.2
**19**	82.7 ± 4.7	10.52 ± 2.69	16.42 ± 0.59	7.9	5.0
**20**	89.9 ± 3.7	7.21 ± 3.52	8.71 ± 0.68	12.5	10.3

^a^ Compound concentration (µM) required to reduce the viability of mock-infected VERO-76 (monkey normal kidney) monolayers by 50%. The results are expressed as CC_50_ ± SD of three different experiments performed in duplicate. ^b^ See Table 1. ^c^ The selectivity index (SI) is expressed as the ratio between the CC_50_ value of each compound against Vero-76 cell line and the IC_50_ of each compound against *L. tropica* or *L. infantum* promastigotes.

**Table 3:** In vitro antileishmanial activity against intramacrophagic amastigotes of *L. infantum* and cytotoxicity against PMA-differentiated THP-1 (human acute monocytic leukemia cell line).

Compd.	*L. infantum* Amastigotes IC_50_^a^ (µM)	THP-1 CC_50_^a,b^ (µM)	SI ^c^
**1**	3.49 ± 0.18	27.97 ± 8.00	8.0
**14**	2.13 ± 1.35	21.66 ± 8.83	10.2
**17**	2.70 ± 0.68	53.90 ± 3.98	20.0
**Lucanthone**	3.43 ± 0.93	15.17 ± 0.27	4.4

^a^ Data are expressed as mean ± SD of three experiments in triplicate. ^b^ Compound concentration (µM) required to reduce the viability of PMA-differentiated THP-1 by 50%. ^c^ The selectivity index (SI) is expressed as the ratio between the CC_50_ value of each compound against THP-1 cell line and the IC_50_ against *L. infantum* amastigotes.

## Supplementary Materials

The following are available online at https://www.mdpi.com/1424-8247/13/11/339/s1. Table S1: In silico binding thermodynamics of compounds **14**, **17**, lucanthone and amitriptyline towards TryR. Table S2: Per-residue binding enthalpy decomposition (ΔH_res_) for compounds **14**, **17**, lucanthone and amitriptyline towards TryR, Figure S1: Amitriptyline in the binding pocket of TryR.
