# Supplementary material for: Quinolizidine-Derived Lucanthone and Amitriptyline Analogues Endowed with Potent Antileishmanial Activity
Source: Pharmaceuticals (Basel). 2020 Oct 25;13(11):339. doi: 10.3390/ph13110339 (PMC7694037; doi:10.3390/ph13110339)
Supplement: Supplementary file 1 [file pharmaceuticals-13-00339-s001.pdf]

Supporting information

Michele Tonelli [1,*], Anna Sparatore [2,*], Nicoletta Basilico [3,*], Loredana Cavicchini [3], Silvia Parapini [4], Bruno Tasso [1], Erik Laurini [5], Sabrina Pricl [5,6], Vito Boido [1] and Fabio Sparatore [1]

Table S1. In silico binding thermodynamics of compounds **14**, **17**, Lucanthone, and Amitriptyline towards TryR. Errors on ΔG_{bind}, ΔH_{bind}, and $T\Delta S_{bind}$ values are within 5%.

Compounds	ΔG_{bind} (kcal/mol)	ΔH_{bind} (kcal/mol)	$-T\Delta S_{bind}$ (kcal/mol)
**14**	-8.73	-24.69	15.96
**17**	-8.54	-23.32	14.78
**Lucanthone**	-8.45	-23.14	14.69
**Amitriptyline**	-7.46	-21.56	14.10

Table S2. Per-residue binding enthalpy decomposition (ΔH_{res}) for compounds **14**, **17**, Lucanthone, and Amitriptyline towards TryR. Only those amino acids critical for the binding are listed. Errors on ΔH_{res} values are within 5%.

Compounds	ΔH_{res} (kcal/mol)		
	E466′, T470′	E18, W21, I339, N340, A343	L17, I106, Y110
**14**	-4.54	-6.86	-3.07
**17**	-4.42	-6.31	-3.11
**Lucanthone**	-4.33	-6.01	-3.22
**Amitriptyline**	-3.58	-5.20	-2.78

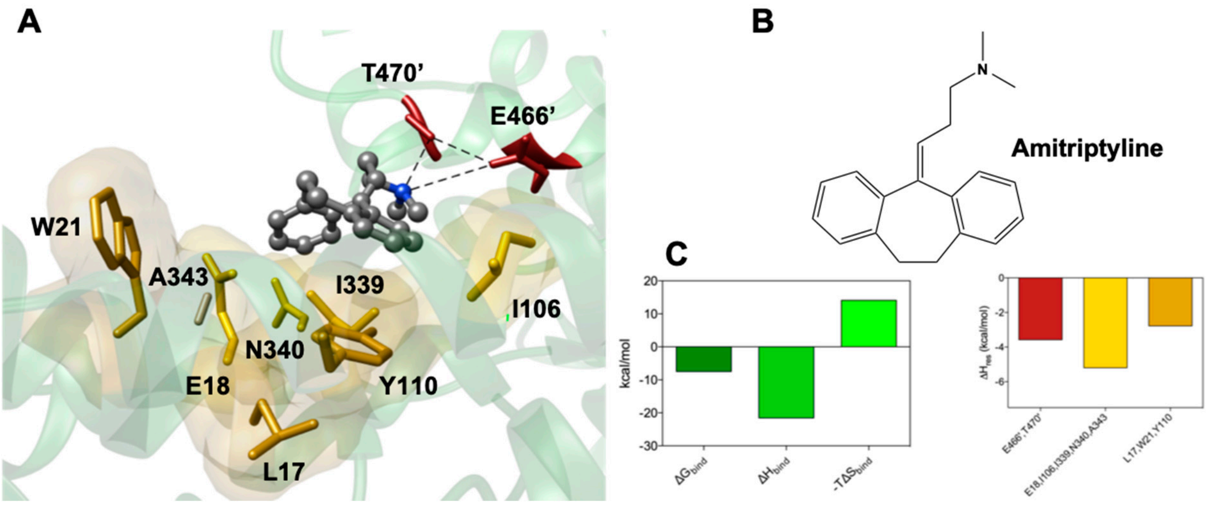

Figure S1: (A) Details of Amitriptyline in the binding pocket of TryR. Compounds are shown as atom-colored sticks-and-balls (C, grey, N, blue, O, red). The side chains of the mainly interacting TryR residues are depicted as colored sticks and labeled as following: E466′ and T470′, firebrick; E18, W21, I339, N340 and A343, gold; L17, I106 and Y110, goldenrod. The hydrophobic pockets are also highlighted by their transparent van der Waals surface. Hydrogen atoms, water molecules, ions, and counterions are omitted for clarity. (B) Molecular structure of Amitriptyline (C) Calculated free energy of binding (ΔG_{bind}, forest green), and enthalpic (ΔH_{bind}, lime green) and entropic ($-T\Delta S_{bind}$, chartreuse) components (left) and PRBFED of the main involved amino acids (right) of TryR in complex with Amitriptyline.
